# The Possible Role of Brain-derived Neurotrophic Factor in Epilepsy

**DOI:** 10.1007/s11064-023-04064-x

**Published:** 2023-11-25

**Authors:** Raed AlRuwaili, Hayder M. Al-kuraishy, Ali I. Al-Gareeb, Naif H. Ali, Athanasios Alexiou, Marios Papadakis, Hebatallah M. Saad, Gaber El-Saber Batiha

**Affiliations:** 1 Department of Internal Medicine, College of Medicine, Jouf University, Sakaka, Saudi Arabia; 2 Department of Clinical Pharmacology and Medicine, College of Medicine, ALmustansiriyia University, P.O. Box 14132, Baghdad, Iraq; 3 Department of Internal Medicine, Medical College, Najran University, Najran, Saudi Arabia; 4 University Centre for Research & Development, Chandigarh University, Chandigarh-Ludhiana Highway, Mohali, Punjab India; 5 Department of Research & Development, Funogen, Athens, Greece; 6 Department of Research & Development, AFNP Med, Wien, 1030 Austria; 7 Department of Science and Engineering, Novel Global Community Educational Foundation, Hebersham, NSW 2770 Australia; 8 Department of Surgery II, University Hospital Witten-Herdecke, University of Witten-Herdecke, Heusnerstrasse 40, 42283 Wuppertal, Germany; 9 Department of Pathology, Faculty of Veterinary Medicine, Matrouh University, Matrouh, 51744 Egypt; 10 Department of Pharmacology and Therapeutics, Faculty of Veterinary Medicine, Damanhour University, Damanhour, AlBeheira, 22511 Egypt

**Keywords:** Epilepsy, Seizure, Brain-derived neurotrophic factor, mTOR pathway, TrkB, Progranulin and α-synuclein

## Abstract

Epilepsy is a neurological disease characterized by repeated seizures. Despite of that the brain-derived neurotrophic factor (BDNF) is implicated in the pathogenesis of epileptogenesis and epilepsy, BDNF may have a neuroprotective effect against epilepsy. Thus, the goal of the present review was to highlight the protective and detrimental roles of BDNF in epilepsy. In this review, we also try to find the relation of BDNF with other signaling pathways and cellular processes including autophagy, mTOR pathway, progranulin (PGN), and α-Synuclein (α-Syn) which negatively and positively regulate BDNF/tyrosine kinase receptor B (TrkB) signaling pathway. Therefore, the assessment of BDNF levels in epilepsy should be related to other neuronal signaling pathways and types of epilepsy in both preclinical and clinical studies. In conclusion, there is a strong controversy concerning the potential role of BDNF in epilepsy. Therefore, preclinical, molecular, and clinical studies are warranted in this regard.

## Introduction

Epilepsy is a protracted neurological disease characterized by repetitive seizures which is hypersynchronous neuronal discharge from explicit brain regions [1]. Epilepsy affects about 1% of the general population worldwide [2]. It has been reported that 80% of epilepsy is more common in developing countries. Epilepsy is more common in the elderly, as 5–10% of old people have seizures at the age of 80 years which augment the chance of a second seizure by more than 40% [3]. One attack of seizure is not epilepsy, but investigations are sensible to detect the underlying causes of the seizure [4]. A history of more than two seizures is diagnostic for epilepsy [1].

**Fig. 1:**
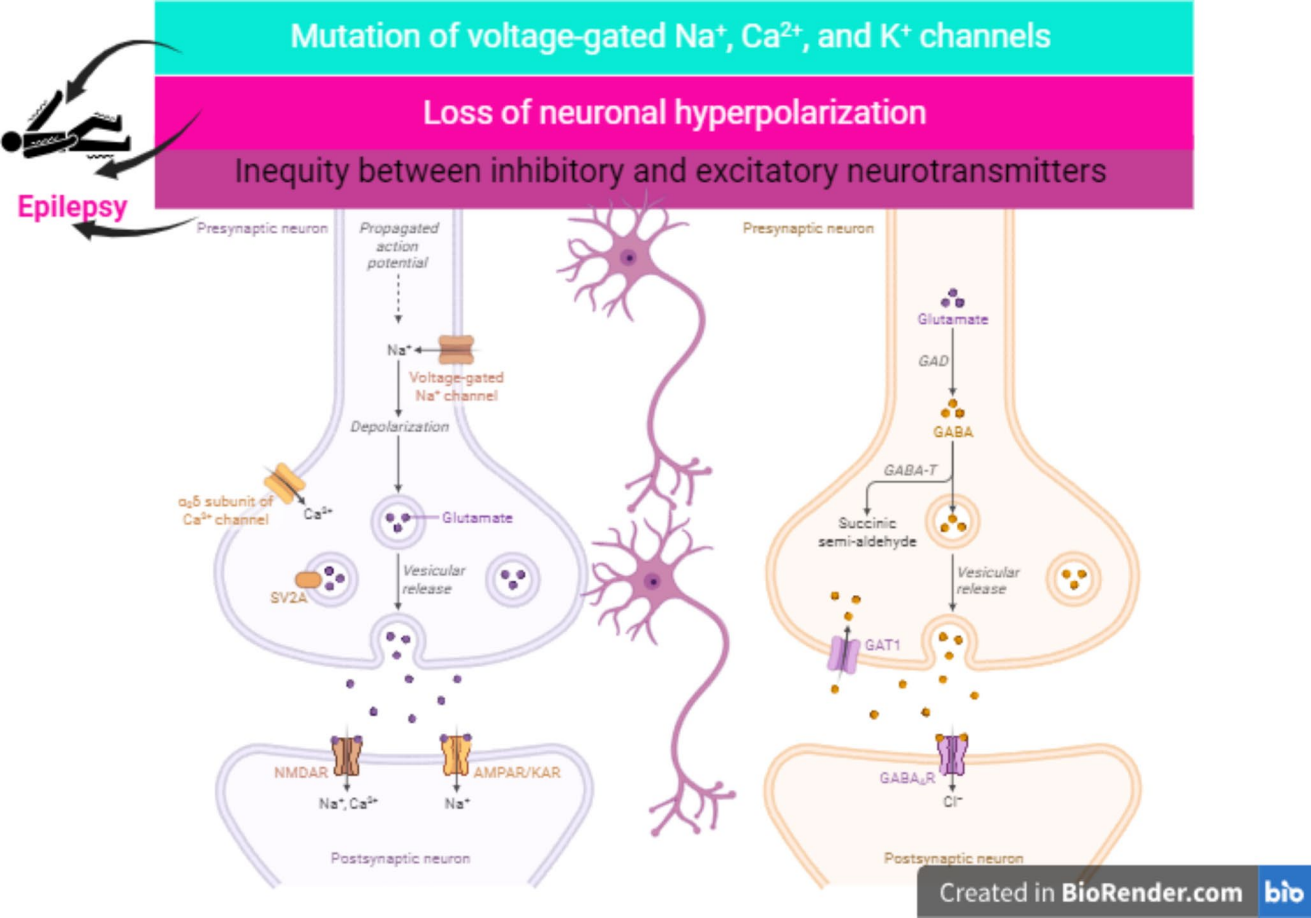
Pathophysiology of epilepsy

The fundamental mechanism of epileptic seizure is due to the development and progression of the epileptogenesis process, and the imbalance between inhibitory and excitatory neurotransmitters and pathways [5]. Reducing inhibitory gamma-aminobutyric acid (GABA) and/or augmentation of excitatory glutamate neurotransmission prompt epileptogenesis [5]. Epileptogenesis is the genesis of a chronic hyperexcitable epileptic state. Epileptogenesis is one of the most dramatic examples of neuronal plasticity, as can be seen by the development of a normal, non-hyperexcitable nervous system into one capable of producing seizures. Epileptogenesis also has many mechanistic similarities with long-term potentiation (LTP) [5]. The causes of epileptogenesis are due to the mutation of voltage-gated Na^+^, Ca^2+^, and K^+^ channels which augment neuronal hyper-excitability and decrease seizure threshold [6]. Mutation of the Na^+^ channel gene SCN8A is accompanied by the progress of epileptogenesis (Fig. 1) [6].

According to etiopathology, two types of epilepsy are known, primary, or idiopathic epilepsy without identified causes. However, secondary epilepsy is caused by diverse causes such as head trauma, tumors, brain infection and neurodegenerative disorders [7]. In this state, different studies indicated that dysregulation of brain-derived neurotrophic factor (BDNF) is concerned with the pathogenesis of epilepsy [8, 9]. However, the molecular mechanisms connecting BDNF with epileptogenesis and epilepsy are not well elucidated. Therefore, the present review aims to revise the potential role of BDNF in epilepsy regarding its beneficial and detrimental roles.

## Brain-derived Neurotrophic Factor

BDNF is a member of the neurotrophins protein family that is involved in neuronal regulation and memory performance [10]. BDNF is the most prevalent growth factor in the central nervous system (CNS), it is vital for the development of the CNS and for neuronal plasticity. Because BDNF plays a crucial role in the development and plasticity of the brain, it is widely implicated in different neuropsychiatric diseases [10]. It has been illustrated that BDNF acts on tyrosine kinase receptor B (TrkB) and p75NT receptor (p75NTR) [11]. BDNF is released from peripheral tissues and the CNS chiefly from the hypothalamus, hippocampus and limbic system [10, 11]. The peripheral action of BDNF is largely related to the modulation of insulin sensitivity and glucose homeostasis [12]. Therefore, BDNF is viewed as metabokine due to its multiple effects on glucose metabolism, blood lipid and other metabolic parameters [13]. Indeed, peripheral and central BDNF levels are reduced by the effects of chronic stress, aging and neurodegenerative diseases [14]. Conversely, exercise and antidepressant agents improve the peripheral and central BDNF levels [15]. Diverse environmental stimuli, such as physical and learning exercises or stress exposure, lead to the activation of specific neuronal networks. These processes require tight temporal and spatial transcriptional control of numerous BDNF splice variants through epigenetic mechanisms which are a cellular process that control gene expression without gene mutations. The dynamic and long-term epigenetic programming of BDNF gene expression by the DNA methylation, histone-modifying and microRNA machineries induce the activity-dependent BDNF mRNA operating critically for rapid local regulation of BDNF levels and synaptic plasticity [16, 17].

Furthermore, BDNF is implicated in the pathogenesis of diverse neurological diseases including Parkinson’s disease (PD), Alzheimer’s disease (AD), multiple sclerosis (MS), amyotrophic lateral sclerosis (ALS) and depression [18]. In addition, impairment of BDNF synthesis is a possible hallmark of numerous neurodegenerative diseases [19]. Selective reduction of BDNF synthesis is extremely reduced in neurofibrillary tangles (NFTs) in AD and in α-synuclein (α-Syn) and Lewy bodies in PD [20, 21]. A clinical study included patients with PD, AD, Lewy body dementia, vascular dementia and frontotemporal dementia and displayed that BDNF serum levels were dysregulated in those patients compared to healthy controls [18]. BDNF serum level is increased in PD patients but reduced in patients with AD, Lewy body dementia, vascular dementia and frontotemporal dementia [18]. Therefore, BDNF serum level is functionally altered in different neurodegenerative diseases [19, 22]. Of note, peripheral BDNF is mainly derived from platelets, vascular and epithelial cells, leukocytes and macrophages [23] that can cross BBB and contribute to brain regulation [24]. Hence, there is a connection between central and peripheral BDNF, and that serum BDNF may reflect brain disorders. Many studies illustrated a positive correlation between central and peripheral BDNF in healthy subjects according to neuroimaging and neuropsychological studies [25, 26]. Underneath this concept, several studies exposed that serum BDNF is reduced in diverse neuropsychiatric diseases such as depression [27, 28]. Moreover, various medications affect the expression and BDNF serum levels in different neurological disorders. For example, mood-stabilizing drugs such as lithium increase the expression of BDNF [29] while, benzodiazepines reduce BDNF serum levels in patients with schizophrenia [30]. Nevertheless, there is a conflicting result on whether these drugs affect the central or peripheral BDNF, and how long effect is needed to produce this effect [31, 32]. These findings confirmed that BDNF is dysregulated in neurodegenerative disorders, and peripheral BDNF levels could reflect the severity and progress of these disorders. Increasing BDNF by different modalities including cognitive stimulation, diet restriction, in vivo and exvivo delivery of BDNF, BDNF mimetics and direct administration of BDNF may improve certain neurological disorders [22, 33]. These data highlighted that BDNF plays a critical role in the modulation of neuronal functions in neurodegenerative and neuropsychiatric diseases. Therefore, targeting of BDNF in those diseases could open new avenue therapeutic modalities in the management of neurodegenerative and neuropsychiatric diseases.

## BDNF and Epilepsy

BDNF is essential for learning and memory by regulating of LTP and synaptic plasticity in the hippocampus [34]. A deficiency of BDNF inhibits hippocampal LTP in mice, and administration of BDNF can restore this defect [35]. LTP is highly related to epileptogenesis and the development of epilepsy [36]. LTP is highly distorted in the early phase of epileptogenesis leading to hyper-excitability and development of epilepsy [36, 37]. Epileptogenesis shares many similarities with long-term potentiation [37].

The mechanisms of BDNF and TrkB expression are controlled by gene regulatory sequences, transcription factors, and epigenetic mechanisms such as DNA methylation and posttranslational modifications of histones [38]. High-order epigenetic mechanisms determining the relationship between the position of the gene within the cell nucleus, and the level of BDNF expression are well recognized [39]. Preclinical findings revealed that BDNF requires > 5 min to begin transcription upon neuronal activation, and persists for several weeks [38]. In addition, epigenetic mechanisms may be intricate in the pathophysiology of epileptic seizures [40]. Therefore, genetic epilepsy markers have been reported to be increased in histone modification markers, indicating the role of epigenetic control of gene transcription in epilepsy pathogenesis [40]. BDNF which has been suggested to lead to seizure-induced pathological processes in the hippocampus is among the numerous genes with expression alterations after seizures. Also, seizure-induced BDNF mRNA downregulation is triggered by histone deacetylase, though histone deacetylase inhibitors prevent both BDNF-associated histone deacetylation and BDNF mRNA downregulation following seizures [41].

Consequently, BDNF is involved in epileptogenesis and epilepsy that could be protective or detrimental. However, the precise mechanism and role of BDNF in epilepsy is still controversial.

### Protective Effect of BDNF in Epilepsy: NO

#### Preclinical Findings

BDNF is concerned with the pathogenesis of epileptogenesis by augmenting neuronal excitability. Expression of BDNF mRNA is correlated with seizure activity and epileptogenesis [42]. It has been shown that BDNF is highly expressed in brain regions that are implicated in the pathogenesis of epilepsy and epileptogenesis [42]. Thus, interfering with neuronal BDNF signaling and transduction could be a new target against epilepsy and epileptogenesis. It has been revealed that BDNF has an excitatory role on animal brain slices and cultured neurons [43]. BDNF/TrkB axis is highly upregulated in the hippocampus in animal model epilepsy [43]. Acute intra-cerebral administration of BDNF induces the development and progression of seizures in mice [43]. Augmentation expression of BDNF in astrocytes also exaggerates pilocarpine-induced seizure in mice. Originally, genetic deletion of BDNF or TrkB in astrocytes attenuates neuronal firing in vitro models of temporal lobe epilepsy (TLE) [44]. This finding proposed that BDNF/TrkB is intricate in the pathogenesis of TLE. Evidence from in vitro studies indicated that BDNF increases neuronal activity which causes a progressive increase in the expression of BDNF [44]. A progressive increase in BDNF induces ectopic neurogenesis and increases neuronal excitability which is intricate in epileptogenesis and TLE [45]. In relation to axonal growth and formation of mossy fiber sprouting which is linked with the development of TLE, BDNF has been reported to increase mossy fiber sprouting [46] nevertheless other studies did not find this effect [47]. Furthermore, BDNF is involved in sprouting consequences in epilepsy [41].

Different preclinical studies highlighted that BDNF expression in the piriform cortex, entorhinal cortex, and amygdala is increased following seizure [48–50]. In addition, TrkB expression is also increased after a seizure [51]. Depending on findings seizure promotes the expression of BDNF and TrkB, which in turn BDNF provokes seizure activity. Therefore, brain insults such as febrile seizure and encephalitis which induce the expression of BDNF trigger epileptogenesis by altering neuronal circuits in many brain areas, and can induce epilepsy [52]. Similarly, an increase in peripheral BDNF by infection and immune deregulation is also involved in the induction of epileptogenesis [53]. However, the direct involvement of BDNF in epileptogenesis by increasing glutamatergic and reducing GABAergic neurotransmission was suggested by different studies [42, 43, 54, 55]. It has been shown that expression of BDNF and TrkB was increased in mice with epilepsy [56, 57]. TrkB over-expression is associated with inhibition of GABAergic neurotransmission, and inhibition of TrkB reduced epileptogenesis in mice [57]. Increasing BDNF and TrkB after seizure induces downregulation of chloride ion cotransporter (KCC2) on GABAergic neurons leading to hyper-excitability and recurrent seizure in mice [58]. Of interest, pro-BDNF release and p75NT expression are augmented in experimental epilepsy due to the inhibition of machinery cleavage of pro-BDNF to BDNF [44]. Plasminogen proteolytic activity which is involved in the cleavage of pro-BDNF to BDNF is highly reduced following seizure and status epilepticus (SE) [59]. It has been shown that a deficiency of plasminogen activator and its receptors promotes seizure in mice [59]. Increasing pro-BDNF and expression of its receptor p75NT after SE triggers downregulation of KCC2 and dysregulation of chloride homeostasis in GABAergic neurons leading to hyper-excitability and epileptogenesis [60, 61]. Blocking of p75NT by a selective antagonist attenuates seizure severity and frequency by restoring the activity and expression of KCC2 [60, 61].

Furthermore, the deletion of TrkB in animal model study eliminates epileptogenesis while activation of TrkB by estrogen triggers epileptogenesis in female rats [62]. Consequently, BDNF/TrkB signaling is involved in the development and progression of epilepsy. Likewise, selective TrkB antagonist attenuates the development of TLE in mice [56]. As well, BDNF provokes seizure in mice through the induction of apoptosis and neuronal injury in the hippocampus [63]. Of interest, BDNF promotes oxidative stress and mitochondrial dysfunction which trigger the development and progression of epilepsy [64, 65]. These preclinical data pointed out that BDNF has detrimental effects by enhancing epileptogenesis in animal model studies.

#### Clinical Findings

**Fig. 2:**
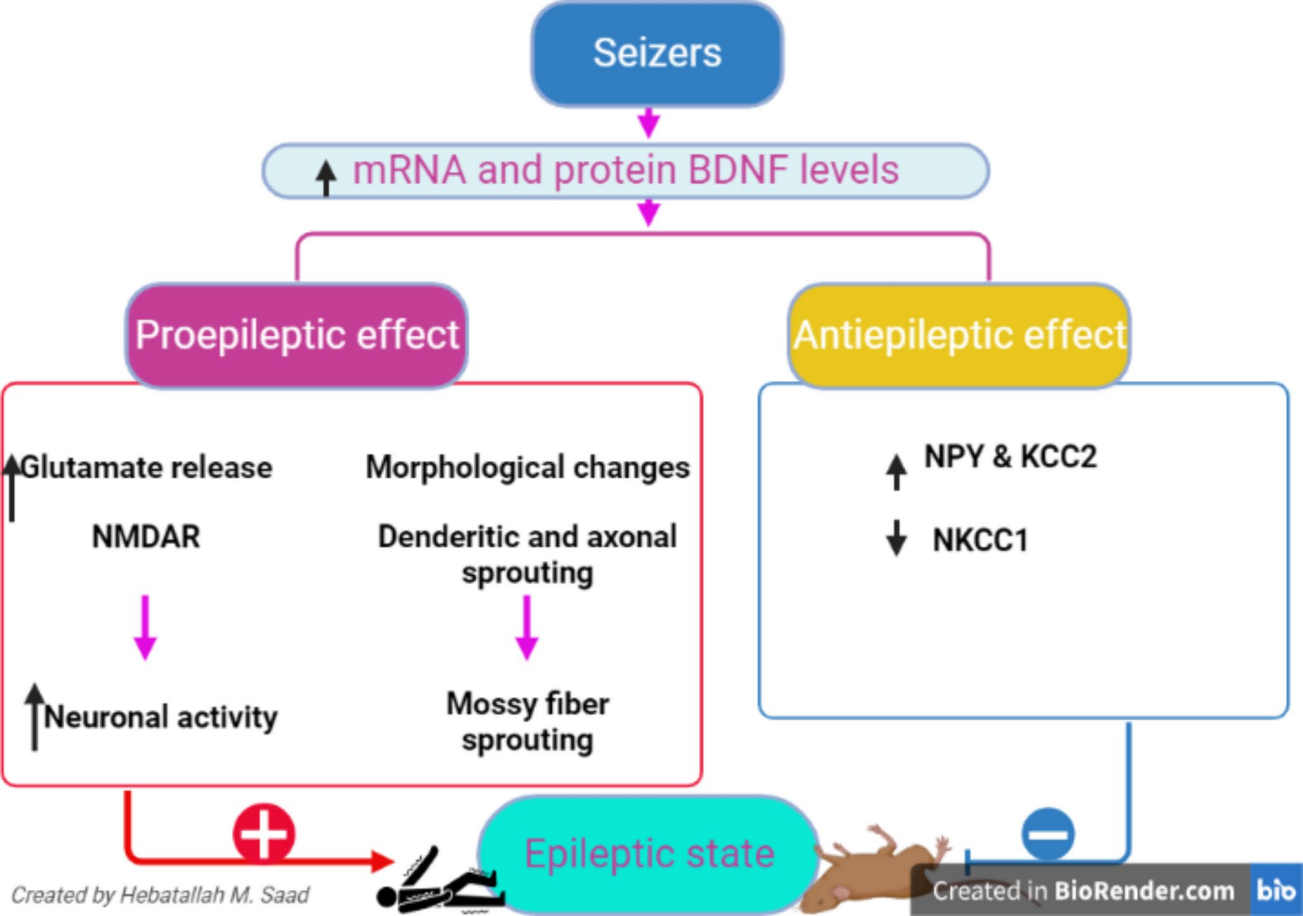
Effect of epileptic seizure on BDNF

In normal physiological conditions, BDNF supports neuronal function and synaptic plasticity [66]. However, increasing BDNF levels may play a contrasting effect by increasing neuronal excitability, neuronal injury and predisposes for the development of epilepsy [67]. BDNF serum levels and TrkB expression are correlated with severity in patients with TLE [43]. Higher expression of BDNF was recently confirmed to be associated with seizure severity in epileptic patients [68]. However, patients having polymorphism of the BDNF gene, and carriers are less susceptible to seizure in Rett syndrome [69]. Conversely, polymorphism of BDNF in the Asian population is correlated with the incidence of epilepsy [70]. A case-control study on 80 patients with epilepsy and 13 healthy controls revealed that BDNF serum levels were higher in epileptic patients as compared to controls [71]. Furthermore, BDNF serum level is higher in epileptic patients and connected with disease severity mainly in TLE [43]. A large cohort study involved 446 epileptic patients and 166 healthy controls illustrated that BDNF serum level was increased in epileptic patients as compared to controls [72]. A systematic review demonstrated that BDNF/TrkB is overstated in epilepsy and associated with seizure severity [43]. Therefore, inhibition of BDNF/TrkB signaling and induction of neuropeptide Y (NPY) expression may reduce seizure frequency and severity in patients with epilepsy. Surgical resection of the hippocampus from patients with TLE revealed that BDNF expression was highly expressed in the hippocampus. Quantitative estimation of mRNA levels in the hippocampus of patients with resistance epilepsy compared to autopsy controls showed increased mRNA hybridization of BDNF with significant reduction of mRNA hybridization of Ca^+ 2^/calmodulin-dependent protein kinase II [73]. This finding suggests that chronic epilepsy and hippocampus epileptic activity triggers significant changes in the gene expression of BDNF. Interestingly, increasing neuronal BDNF expression is correlated with the development of TLE which developed due to disruption of the balance between inhibitory GABA and excitatory glutamate [54]. A previous cohort study indicated that BDNF serum level was increased in patients with TLE [74]. TLE is the most resistant type of epilepsy to the effect of AEDs [75]. BDNF modifies neuronal synapses and synaptic plasticity in both adult and developing brains [55]. In chronic epilepsy mainly TLE, BDNF is up-regulated leading to disruption the inhibitory and excitatory neuronal signaling pathway causing seizure [54, 55]. BDNF increases excitatory glutamate and reduces inhibitory GABA with the induction of seizure [54]. KCC2 expression is reduced in patients with TLE due to dysregulation of pro-BDNF/BDNF ratio [76]. In addition, epileptic seizure and SE induces dysregulation of pro-BDNF/BDNF axis with subsequent downregulation regulation of KCC2 expression leading to recurrent seizure [62] (Fig. 2).

Of note, BDNF gene expression in the temporal cortex and hippocampus is augmented in patients with TLE [68]. However, it is still unclear whether the stimulatory effect of BDNF is through presynaptic activation releases of glutamate or phosphorylation of postsynaptic GABA receptors [43]. These clinical findings indicated that an exaggerated BDNF level is associated with epilepsy. However, there is no clinical study that measures BDNF levels in both serum and CSF at the time of seizure due to ethical limitations.

### Protective Effect of BDNF in Epilepsy: YES

#### Preclinical Findings

It has been displayed that BDNF may be beneficial against seizure progression by enhancing the inhibitory GABAergic neurotransmission [77, 78]. Chronic treatment with BDNF inhibits seizure severity and frequency following induction of SE in the animal model study [77]. BDNF can induce LTP of GABAergic neurons, prevent internalization of GABA receptors via activation of protein kinase and inhibit the interaction with phosphatase 2 A complex downstream of protein kinase [78]. BDNF decreases neuronal excitability by increasing NPY [43]. NPY is considered an endogenous anti-seizure via activation of Y2-Y5 receptors expressed in neurons [79]. Therefore, NPY-based gene therapy may be a new anti-epileptic agent for the management of resistance epilepsy. BDNF is reduced in adult epileptic patients [80]. Indeed, TrkB agonists prevent post-traumatic epilepsy by inhibiting epileptogenesis [81]. Chronic infusion of BDNF in mice reduces neuronal excitability by downregulating TrkB and increases the expression of neuroprotective NPY [43].

Certainly, BDNF/TrkB role is differed according to specific brain regions, it reduces neuronal excitability in the neocortex but augments neuronal excitability in the hippocampus [77]. Furthermore, continuous administration of BDNF by a bio-delivery system attenuates generalized epilepsy in rats [82]. Thus, BDNF/TrkB signaling seems to be beneficial rather than harmful in epilepsy, and increasing BDNF level in epilepsy could be a compensatory mechanism to prevent seizure-induced neuronal injury [83]. It has been shown that BDNF which binds TrkB and pro-BDNF which binds p75NTR receptors are involved in the repair of neuronal injury and regulation of synaptic plasticity [38]. Various preclinical studies highlighted the protective role of epileptogenesis and the development of epilepsy. BDNF inhibits epileptogenesis by reducing neuronal excitability in the pyramidal neurons [84]. BDNF improves GABAergic neurotransmission in rats with experimental epilepsy through the phosphorylation of different subunits [85]. Deletion of BDNF/TrkB induces hyper-reactivity of interneurons with the development of seizures in mice [86]. TrkB through different molecular effects improves the maturation of GABAergic and inhibitory interneurons, and loss of BDNF disturbs the balance of the inhibitory/excitatory axis [87]. Furthermore, BDNF can reduce epileptogenesis-induced inflammation through the improvement of BBB integrity in rats with experimental epilepsy [88]. BDNF has a neuroprotective effect and inhibits epileptogenesis thereby reducing epilepsy induced neuronal injury in rats [89]. Supporting this notion, an experimental study illustrated that a selective α-2 agonist dexmedetomidine attenuates kainic acid-induced seizure in rats model of TLE by increasing the expression of TrkB and release of BDNF [90]. Dexmedetomidine inhibits neuronal glutamate release and can reduce excitotoxicity-induced neuronal injury by modulating inflammatory signaling pathways and BDNF [90]. Therefore, dexmedetomidine could be a potential anti-epileptic agent. Likewise, a proton-pump inhibitor pantoprazole has been recently shown to attenuate PTZ-induced seizure in rats by increasing the expression of brain BDNF [91]. Moreover, a recent experimental study demonstrated that probiotics can attenuate PTZ-induced seizure in rats by increasing the expression of BDNF [91]. In vitro study demonstrated that pantoprazole reduces PTZ-induced neurotoxicity in the SH-SY5Y cell line by decreasing oxidative stress and apoptosis with increasing the expression of BDNF [91]. Furthermore, hesperidin protects from PTZ-induced neurotoxicity and epilepsy via stimulation of the cAMP response element binding protein (CREB)/BDNF pathway [92]. TrkB activates the expression of CREB which improves the expression of BDNF that promotes TrkB signaling [82]. Further preclinical studies indicated that the reduction of BDNF increases the risk of epilepsy [82]. Therefore, increasing the expression or delivery of BDNF into the hippocampus which is involved in epileptic activity can decrease the frequency of seizure and reverses different structural neuronal changes linked with chronic epilepsy [82]. In this state, augmentation of BDNF/TrkB could be beneficial in the management of chronic epilepsy. However, there is no solid clinical evidence support that increasing of BDNF above normal physiological level to inhibits epileptogenesis. In addition, the molecular mechanism behind the suppressant effect of BDNF on epileptogenesis still unidentified.

#### Clinical Findings

Diverse clinical studies confirmed that BDNF plays a critical role in preventing epileptic seizures. Of note, BDNF serum level was reduced in patients with TLE as compared to healthy controls [93]. A case-control study on 12 patients with psychogenic non-epileptic seizure (PNES), 15 patients with an epileptic seizure, and 17 healthy controls revealed that BDNF level serum was reduced in patients with epileptic seizure compared to other patients and healthy controls [80]. This study had a small sample size which affects the causal relationship between epilepsy and BDNF serum level. A systematic review demonstrated that BDNF serum levels in epileptic patients was not different compared to the general population [77]. In addition, BDNF serum level is reduced in patients with partial epilepsy [77].

BDNF is mainly expressed in the hippocampus a site of epileptic seizure in TLE. Therefore, decreasing of BDNF circulating levels in patients with TLE indicates impairment of brain white matter and associated cognitive dysfunction [93]. However, this small sample size does not give concrete clinical confirmation concerning the association between low levels of BDNF in patients with TLE. Similarly, AEDs can downregulate BDNF expression leading to the reduction of BDNF serum levels in epileptic patients [94]. A case-control study on 143 epileptic patients compared to 48 healthy control subjects exposed that BDNF serum level was reduced in epileptic patients compared to controls [95]. BDNF serum level was reduced rapidly within 1 h in epileptic patients following acute seizure [95]. The underlying cause for the reduction of BDNF after acute epileptic seizure is not fully elucidated. It has been observed that epileptic seizure induced oxidative stress which causes hippocampal injury and inhibition of neurogenesis [96]. Herein, progressive neuronal injury and dysfunction of hippocampal synaptic plasticity are associated with the reduction of BDNF following seizure [97]. Also, the BDNF gene is downregulated during epileptic seizures [98]. Inhibition of machinery cleavage of pro-BDNF to BDNF and reduction of plasminogen proteolytic activity following seizure and SE could be a possible mechanism for decreasing BDNF in epilepsy [44, 59]. In addition, BDNF gene polymorphism affects the release and functional activity in patients with TLE [99]. Moreover, the BDNF signaling pathway inhibits epileptogenesis by modulating the expression of miR124 which induces neuronal excitability [100]. Induction the release of BDNF by exercise can reduce seizure frequency by promoting cellular signaling pathways which involves reducing neuronal excitability and improving synaptic plasticity [101].

These verdicts indicated that BDNF is highly reduced in epileptic patients. In sum, there is a strong controversy concerning whether BDNF serum levels in epilepsy could be protective or detrimental.

## Discussion

The present review highlighted that BDNF has detrimental and protective effects in relation to the development of epileptogenesis and the progression of epilepsy. In 1995, it was reported that over-expression of BDNF was linked with epileptogenesis [102]. This foundation excited many researchers to illustrate the link between BDNF and epilepsy. Croll and coworkers in 1999 found that BDNF triggered in vitro hyper-excitability and increased seizure severity in mice [103]. In transgenic mice, over-expression of TrkB is associated with seizure frequency and severity [104]. However, the underlying mechanism for BDNF-induced seizure is controversial since both high and low BDNF can interrupt the neuronal inhibitory/excitatory axis through disruption of neuronal LTP [105]. Preclinical studies that measure BDNF in epileptogenesis and induced epilepsy may not accurately reflect the level of endogenous BDNF as it is affected by different factors including age, sex and diurnal variations [106]. Moreover, little is recognized about normal concentration and cut-off values of BDNF and pro-BDNF in healthy and epileptic patients. For example, antibodies against BDNF to localize its main site in the neurons showed controversial findings, as it is highly dense in synapses or transported from soma to the dendritic during seizure [107]. Andreska et al. [108] confirmed by an experimental study that BDNF expression is highly abundant at hippocampal glutamatergic presynapses. Therefore, the excitability of hippocampal glutamatergic neurons in epileptogenesis may be linked with the release of BDNF [109]. Thus, elevation of BDNF following seizure could be a compensatory mechanism to reduce the excitability of hippocampal glutamatergic neurons [110]. BDNF-mediated activation of TrkB exerts different effects on epileptogenesis depending on the types of epilepsy model, natural history of experimental epilepsy, time of administration and TrkB inhibition or activation [111, 112]. BDNF is reduced in partial epilepsy but increased in generalized epilepsy [94]. However, BDNF was found to decrease desensitization of GABA receptors in humans and animals [113, 114]. However, the administration of BDNF in rats’ hippocampus did not affect seizure frequency and severity [115]. Activation of the TrkB receptor in animals with post-traumatic epilepsy hinders epileptogenesis through the modulation of parvalbumin interneurons [81]. However, inhibition of TrkB after SE in animal model studies can attenuate the development of TLE [77]. TrkB antagonists and agonists are not available in clinical practice to modulate the BDNF-induced epileptogenesis. In this manner, BDNF mimetics could be effective in the management of epilepsy by reducing hippocampal neuronal injury [116]. These outcomes indicated conflicting results regarding BDNF effects which might be pro-epileptogenic or anti-epileptogenic.

To understand the exact effect of BDNF in epilepsy, should revise the molecular signaling associated with epilepsy concerning BDNF expression.

### Autophagy and BDNF in Epilepsy

Autophagy is a precise cellular process to eliminate different cytoplasmic misfolded proteins, lipid and damaged organelles to the lysosomes for degradation and clearance [117]. Autophagy role in epilepsy has been lately considered as autophagy inducer rapamycin plays a critical role in the attenuation of induced seizure in animal model studies [118]. Induced autophagy in response to oxidative stress contributes to neuronal cell deaths after seizure in animal model study [119]. Inhibition of oxidative stress by antioxidants significantly attenuates autophagic response in pilocarpine-induced epilepsy [119]. It has been recommended that autophagy prevents the development and progression of epilepsy through regulation the balance between inhibitory GABA and excitatory glutamate [120]. Autophagy is intricate in synaptic homeostasis and the regulation of neurotransmitters, thus imperfect autophagy is associated with reduction the activity of certain neurotransmitters mainly GABAergic ones [121]. In addition, the heterogeneity of GABAergic interneurons affects epileptogenesis and hyper-excitability in epilepsy [122]. Consequently, defective autophagy promotes epileptic seizure in animal and human studies [123, 124]. Induction of autophagy and autophagy-related proteins like Atg7, LC3, and Beclin-1 by endothelial progenitor cells could be a novel therapeutic strategy in the management of epilepsy [9].

It has been reported that BDNF improves synaptic plasticity by inhibiting autophagy which is implicated in the degradation of synaptic proteins [125]. Under the starvation condition, BDNF is activated leading to the activation of the PI3K/Akt pathway which inhibits expression of autophagic protein and the formation of autophagosomes [125]. Conversely, an in vitro study demonstrated that BDNF promotes autophagy by inhibiting PI3K/Akt pathway [126]. Furthermore, an experimental study showed that corticosterone-induced depression is mediated by autophagy hyperactivation and associated reduction of BDNF by excessive lysosomal degradation [127]. Despite these conflicting findings regarding the relationship between autophagy and BDNF, a recent preclinical study confirmed that autophagy improves BDNF signaling [128]. Stress-induced autophagy promotes the release of matrix metalloproteinase 9 (MMP9) which enhances the cleavage of pro-BDNF to BDNF [128]. It has been reported that MMP9 increases availability and optimizes the functional activity of BDNF to facilitate synaptic plasticity and activation of cortical neurons [129]. Notably, MMP9 activity is augmented in the epileptic foci as observed in preclinical and clinical studies [130]. MMP9 triggers BBB injury and neuroinflammation in epilepsy [130]. Therefore, exaggerated autophagy and release of BDNF in epilepsy could explain acceleration of BDNF in relation to epileptogenesis. However, this effect could beneficial rather than detrimental since autophagy inducers like metformin an insulin sensitizing drug used as a first-line in the management of diabetes can reduce seizure severity [131]. Furthermore, metformin attenuates the development of SE by inducing autophagy [131]. A cohort study involved 18 patients with Lafora disease, 8 treated with metformin, and 10 untreated showed that metformin was effective in reducing seizure severity and frequency [132]. Similarly, a macrolide antibiotic rapamycin is effective in patients with tuberous sclerosis complex and could be as an adjuvant treatment with AEDs [133]. Both metformin and rapamycin promote expression of BDNF [134, 135]. Thus, autophagy/BDNF pathway is an essential pathway to maintain neuronal integrity and attenuate epileptogenesis and the development of epilepsy.

### Mechanistic Target of Rapamycin (mTOR) and BDNF in Epilepsy

Importantly, the mTOR pathway is an integral pathway intricate in the regulation of neurogenesis, synaptic plasticity, neuronal development and excitability [136]. It has been perceived that mTOR/autophagy axis controls synaptic plasticity, vesicular release, and clustering of GABA receptors with regulation of inhibitory/excitatory balance in the brain [137]. Overstated mTOR activity is related to the progress of TLE, genetic and acquired epilepsy, experimental epilepsy and Lafora disease [138]. Inhibition of the mTOR pathway reduces seizure severity through the activation of autophagy [139]. Inhibition of mTORpathy according to the findings from preclinical and clinical trials may be effective in the management of genetic and acquired epilepsies [139]. Of note, the mTOR pathway is regarded as a negative regulator of autophagy. Inhibition of the mTOR pathway by metformin and rapamycin may explain the protective role of these agents against epilepsy [140]. It has been observed that BDNF improves memory consolidation through the induction of the mTOR pathway in mice [141]. Inhibition of mTOR pathway by rapamycin in mice with TLE induces activation of BDNF [142]. Likewise, six-week exercise reduces seizure frequency in rats through regulation of the BDNF/ mTOR pathway [143]. An elegant experimental study observed that BDNF activates autophagy by inhibiting the mTOR pathway in rats with hypoxic-ischemic encephalopathy [8]. Furthermore, an exaggerated mTOR pathway in diabetes inhibits BDNF signaling leading to neuroinflammation and synaptic dysfunction in diabetic encephalopathy [144]. Thus, BDNF through inhibition of the mTOR pathway and induction of autophagy could be an effective strategy against epileptogenesis.

### Progranulin and BDNF in Epilepsy

Progranulin (PGN) is a preserved secreted protein expressed by diverse cell types in the CNS and peripheral tissues [145]. PGN switches cell growth and inflammation, lysosomal function and microglial response [145]. In the CNS, PGN is mostly expressed by microglia and induces uptake of synaptophysin by microglia [146]. It has been shown that mutation of PGN is linked with the development of frontotemporal dementia and other neurodegenerative disorders [147]. It has been shown that PGN expression is increased in the hippocampus after status epilepticus in mice as a compensatory mechanism [148]. PGN expression is augmented by macrophages and microglia in the hippocampus, cerebral cortex and thalamus within 48 h following pilocarpine-induced SE [149]. Besides, CSF PGN level was documented to be increased in epileptic patients following SE as compared to control [149]. A cohort study on patients with resistance epilepsy (*n* = 56) exposed that CSF PGN level was increased as compared to healthy (*n* = 36) [150]. Importantly, metformin activates the expression of neuroprotective and anti-inflammatory PGN [138]. Findings from an experimental study showed that pre-treatment with metformin increases PGN which improves anti-inflammatory cytokines and reduces reactive astrogliosis [151]. Deficiency of neuronal PGN due to mutation promotes complement activation which enhances the engulfment of inhibitory synapses by microglia [152]. Consequently, increasing PGN in epilepsy mainly after SE could be a compensatory mechanism to protect inhibitory synapses from injury by microglia. It has been reported that PGN is co-secreted with BDNF, and PGN activates the release of BDNF [153]. PGN acts on specific receptor sortilin-1 which also mediates the function of BDNF. Sortilin-1 regulates BDNF by modulating lysosomal trafficking and anterograde transport. Sortilin-1 forms a complex with pro-BDNF and p75 to promote cell death. As well, sortilin-1 enhances the expression of TrBk on the neuronal terminals [154]. Reduction of PGN is in parallel with reduction of BDNF in different neurodegenerative diseases [155]. Therefore, these findings suggest that increasing of BDNF in epilepsy may due to augmentation the effect of PGN.

### Alpha-synuclein and BDNF in Epilepsy

Synucleins (Syns) are extremely ample proteins in the CNS, that control synaptic vesicle trafficking and neurotransmitter release [156]. α-Syn is intricate in the formation of Lewy bodies a hallmark of PD and other neurodegenerative diseases such as AD [157]. The mechanism of α-Syn-induced neurodegeneration is not thriving assumed [158]. However, the formation of neurotoxic α-Syn filaments could be the conceivable mechanism [158]. Epilepsy and neurodegenerative diseases share a common underlying mechanism [159]. Released α-Syn from injured neurons triggers astrocytes and microglia leading to neuroinflammation and degeneration of inhibitory neurotransmitters with subsequent induction of epileptogenesis [160]. Indeed, α-Syn expression is increased in the hippocampus in rats with PTZ-induced seizure [161]. In addition, α-Syn expression is advanced in epileptic brains as compared to normal brains and is associated with disease severity [161]. Likewise, pilocarin-induced seizure in mice triggers expression of α-Syn in the brain within 4 weeks from induction of epilepsy [162]. In the clinical background, it has been stated that α-Syn expression in the brain of patients with TLE was increased [163]. Serum α-Syn level is augmented in epileptic children interrelated with disease severity and cognitive dysfunction [164]. Relevant, serum α-Syn level is linked with CSF α-Syn level and IL-6 [160]. Serum and CSF α-Syn levels are increased in patients with refractory epilepsy [165]. These outcomes provide evidence that epilepsy is linked with neurodegenerative disorders, and α-Syn serum level could be a diagnostic and prognostic biomarker of refractory epilepsy. Therefore, targeting of α-Syn may reduce epileptogenesis in patients with neurodegenerative disorders and epilepsy [159].

Regarding the relationship between α-Syn and BDNF, it has been observed that α-Syn inhibits the expression of BDNF through inhibition of cAMP and CREB [166]. A preclinical study conducted by Feng et al. [167] revealed that accumulation of α-Syn in mice PD model causes neuronal injury and reduction of circulating BDNF. Activation of BDNF attenuates the accumulation of α-Syn in mice [168]. Of interest, a wild-type α-Syn triggers activation of BDNF, though mutant α-Syn suppresses BDNF [21]. Furthermore, α-Syn attenuates the functional activity of TrkB in the PD model [169]. Of note, an MAO-B inhibitor rasagilin prevents the interaction between α-Syn and TrkB in the PD model with subsequent rescuing of the BDNF/TrkB signaling pathway [169]. Higher expression of α-Syn in intractable epilepsy [165] could explain the reduction of circulating BDNF in patients with severe and resistant epilepsy.

Taken together, the potential role of BDNF in epilepsy could detrimental by interrupting the neuronal inhibitory/excitatory axis, or beneficial by inhibiting the excitability of hippocampal glutamatergic neurons. However, dysregulation of BDNF in epilepsy is not a single entity but is related to the dysregulation of autophagy, mTOR pathway, PGN and α-Syn which negatively and positively regulate the BDNF/TrkB signaling pathway. Therefore, the measurement of BDNF in epilepsy should be related to other neuronal signaling pathways and types of epilepsy in both preclinical and clinical studies.

## Conclusions

Epilepsy is a neurological disease characterized by repeated seizures. BDNF is increased or decreased in epilepsy, and depending on these findings, BDNF is implicated in epileptogenesis and epilepsy. However, BDNF may have a neuroprotective effect against epilepsy. Thus, the goal of the present review was to highlight the protective and detrimental roles of BDNF. Preclinical and clinical data illustrated that BDNF has detrimental effects by enhancing epileptogenesis. However, other findings indicated that BDNF has protective effects against epileptogenesis. The autophagy/BDNF pathway is an essential pathway to maintain neuronal integrity and attenuate epileptogenesis and the development of epilepsy. BDNF through inhibition of the mTOR pathway and induction of autophagy could be an effective strategy against epileptogenesis. The increasing BDNF in epilepsy may be due to augmentation of the effect of PGN. Furthermore, higher expression of α-Syn in intractable epilepsy could explain the reduction of circulating BDNF in patients with severe and resistant epilepsy. These outcomes excite many researchers to illustrate the link between BDNF and epilepsy by preclinical and clinical studies.

## Data Availability

Data sharing is not applicable to this article as no new data were created or analyzed in this study.
