# Correction to: The Possible Role of Brain-derived Neurotrophic Factor in Epilepsy

**DOI:** 10.1007/s11064-023-04092-7

**Published:** 2024-01-06

**Authors:** Raed AlRuwaili, Hayder M. Al-kuraishy, Ali I. Al-Gareeb, Naif H. Ali, Athanasios Alexiou, Marios Papadakis, Hebatallah M. Saad, Gaber El-Saber Batiha

**Affiliations:** 1 Department of Internal Medicine, College of Medicine, Jouf University, Sakaka, Saudi Arabia; 2 Department of Clinical Pharmacology and Medicine, College of Medicine, ALmustansiriyia University, P.O. Box 14132, Baghdad, Iraq; 3 Department of Internal Medicine, Medical College, Najran University, Najran, Saudi Arabia; 4 University Centre for Research & Development, Chandigarh University, Chandigarh-Ludhiana Highway, Mohali, Punjab India; 5 Department of Research & Development, Funogen, Athens, Greece; 6 Department of Research & Development, AFNP Med, 1030 Vienna, Austria; 7 Department of Science and Engineering, Novel Global Community Educational Foundation, Hebersham, NSW 2770 Australia; 8 Department of Surgery II, University Hospital Witten-Herdecke, Heusnerstrasse 40, University of Witten-Herdecke, 42283 Wuppertal, Germany; 9 Department of Pathology, Faculty of Veterinary Medicine, Matrouh University, Matrouh, 51744 Matrouh Egypt; 10 Department of Pharmacology and Therapeutics, Faculty of Veterinary Medicine, Damanhour University, Damanhour, 22511 AlBeheira Egypt

**Correction to: Neurochemical Research** 10.1007/s11064-023-04064-x

In this original article the affiliation details for

1. Raed AlRuwaili were incorrectly given as “University Centre for Research & Development, Chandigarh University, Chandigarh-Ludhiana Highway, Mohali, Punjab, India” but should have been “Department of Internal Medicine, College of Medicine, Jouf University, Sakaka, Saudi Arabia, Raed-123@hotmail.com”
2. Hayder M. Al-kuraishy (haydermutter@uomustansiriyah.edu.iq) and Ali I. Al-Gareeb (Dr.alialgareeb78@yahoo.com) were incorrectly given as “Department of Research & Development, Funogen, Athens, Greece” but should have been “Department of Clinical Pharmacology and Medicine, College of Medicine, ALmustansiriyia University, P.O. Box 14132, Baghdad”.
3. Naif H. Ali were incorrectly given as “Department of Research & Development, AFNP Med, Wien 1030, Austria” but should have been “Department of Internal Medicine, Medical College, Najran University, Najran, Saudi Arabia, Dr.naif1989@gmail.com”.
4. The abbreviated affiliation of “Department of Research & Development, AFNP Med, Wien, 1030, Austria” was also displayed as “AFNP Med, Wien, 1030, Austria”

The original article has been corrected.

