# Editorial Expression of Concern: The Possible Role of Brain-derived Neurotrophic Factor in Epilepsy

**DOI:** 10.1007/s11064-024-04249-y

**Published:** 2024-09-27

**Authors:** Raed AlRuwaili, Hayder M. Al-kuraishy, Ali I. Al-Gareeb, Naif H. Ali, Athanasios Alexiou, Marios Papadakis, Hebatallah M. Saad, Gaber El-Saber Batiha

**Affiliations:** 1 Department of Internal Medicine, College of Medicine, Jouf University, Sakaka, Saudi Arabia; 2 Department of Clinical Pharmacology and Medicine, College of Medicine, ALmustansiriyia University, P.O. Box 14132, Baghdad, Iraq; 3 Department of Internal Medicine, Medical College, Najran University, Najran, Saudi Arabia; 4 University Centre for Research & Development, Chandigarh University, Chandigarh-Ludhiana Highway, Mohali, Punjab India; 5 Department of Research & Development, Funogen, Athens, Greece; 6 Department of Research & Development, AFNP Med, Wien, 1030 Austria; 7 Department of Science and Engineering, Novel Global Community Educational Foundation, Hebersham, NSW 2770 Australia; 8 Department of Surgery II, University Hospital Witten-Herdecke, University of Witten-Herdecke, Heusnerstrasse 40, 42283 Wuppertal, Germany; 9 Department of Pathology, Faculty of Veterinary Medicine, Matrouh University, Matrouh, 51744 Egypt; 10 Department of Pharmacology and Therapeutics, Faculty of Veterinary Medicine, Damanhour University, Damanhour, 22511 AlBeheira Egypt

**Neurochemical Research (2023) 49:533–547**

10.1007/s11064-023-04064-x

The Editor-in-Chief is issuing an Editorial Expression of Concern to alert readers that this article underwent unusual changes to the authorship list during the submission process. Concerns have also been raised about discrepancies between the text of this article and several cited references, including references [2], [91], and [148]. Readers are advised to interpret the findings of this review and the contributions of the authors to this article with caution.

Raed AlRuwaili, Hayder M. Al-kuraishy, Naif H. Ali, Athanasios Alexiou, Hebatallah M. Saad, and Gaber El-Saber Batiha disagree with this Editorial Expression of Concern. Marios Papadakis did not respond to correspondence from the Publisher about this Editorial Expression of Concern. The Publisher was unable to confirm a current contact address for Ali I. Al-Gareeb.

